# Pathway-Based Analysis Using Genome-wide Association Data from a Korean Non-Small Cell Lung Cancer Study

**DOI:** 10.1371/journal.pone.0065396

**Published:** 2013-06-06

**Authors:** Donghoon Lee, Geon Kook Lee, Kyong-Ah Yoon, Jin Soo Lee

**Affiliations:** Lung Cancer Branch, Research Institute and Hospital, National Cancer Center, Gyeonggi, Republic of Korea; Queen Elizabeth Hospital, Hong Kong

## Abstract

Pathway-based analysis, used in conjunction with genome-wide association study (GWAS) techniques, is a powerful tool to detect subtle but systematic patterns in genome that can help elucidate complex diseases, like cancers. Here, we stepped back from genetic polymorphisms at a single locus and examined how multiple association signals can be orchestrated to find pathways related to lung cancer susceptibility. We used single-nucleotide polymorphism (SNP) array data from 869 non-small cell lung cancer (NSCLC) cases from a previous GWAS at the National Cancer Center and 1,533 controls from the Korean Association Resource project for the pathway-based analysis. After mapping single-nucleotide polymorphisms to genes, considering their coding region and regulatory elements (±20 kbp), multivariate logistic regression of additive and dominant genetic models were fitted against disease status, with adjustments for age, gender, and smoking status. Pathway statistics were evaluated using Gene Set Enrichment Analysis (GSEA) and Adaptive Rank Truncated Product (ARTP) methods. Among 880 pathways, 11 showed relatively significant statistics compared to our positive controls (P_GSEA_≤0.025, false discovery rate≤0.25). Candidate pathways were validated using the ARTP method and similarities between pathways were computed against each other. The top-ranked pathways were *ABC Transporters* (P_GSEA_<0.001, P_ARTP_ = 0.001), *VEGF Signaling Pathway* (P_GSEA_<0.001, P_ARTP_ = 0.008), *G1/S Check Point* (P_GSEA_ = 0.004, P_ARTP_ = 0.013), and *NRAGE Signals Death through JNK* (P_GSEA_ = 0.006, P_ARTP_ = 0.001). Our results demonstrate that pathway analysis can shed light on post-GWAS research and help identify potential targets for cancer susceptibility.

## Introduction

Lung cancer is one of the leading causes of cancer mortality in Korea and worldwide [1]–[3]. Among the several lung cancer histological types, more than 70% of Korean lung cancers are non-small cell lung cancers (NSCLCs), the leading subtype being adenocarcinoma [4].

Although the causes of the disease may stem from environmental factors, such as carcinogens found in cigarette smoke and the inhalation of toxic chemicals, in efforts to determine the etiology of the disease, researchers have assessed the genetic diversity of individuals. Several genome-wide association studies (GWASs), which focus on scanning for disease-associated SNPs across the entire genome, have successfully demonstrated possible lung cancer susceptibility loci over the last decade. Most of these GWASs were based on European and American populations and notable suspects identified were 5p15 (hTERT-CLPTM1L) [5], [6], 6p21 (BAT3-MSH5) [7], and 15q25 (CHRNA 3–5) [8]–[10]. In previous GWAS involving 1,425 NSCLC patients and 3,011 controls from Korea, we reported that a novel locus, 3q29, and a locus previously reported in subjects of European descent, 5q15, were associated with lung cancer risk in Korean population [11].

Despite the successful identification of these disease susceptibility loci using GWASs, it is believed that they explain only a small proportion of the estimated heritability [12]. GWASs compare half a million to millions of markers at once and variants with modest associations are likely to be neglected after multiple testing correction [13]. By its nature, it is highly unlikely that a single variant is associated with a complex disease like cancer. It is believed that low-penetrance variants throughout the genome will better explain the biology in question [14]. To compensate for the shortcomings of GWAS, instead of relying on a stringent genome-wide significance cutoff, alternative methods to improve power, such as the use of combinations of SNP markers [15]–[20], incorporation of imputed genotypes and linkage information [21]–[23], and, most recently, pathway-based approaches [24] have been developed.

Pathway-based approaches are based on the principle that genes involved in the same functional pathway interact with each other and constitute a network, so that their disease associations are interrelated [25]. Current pathway-based analyses have been inspired mostly from gene expression microarray data analysis. Based on prior biological knowledge, gene set enrichment analysis (GSEA) [26] measures how much association signals are enriched in a defined set of genes. Because GSEA requires microarray data as input, several groups have suggested modifications to the original algorithm to incorporate GWAS data [27]–[30].

**Figure 1:**
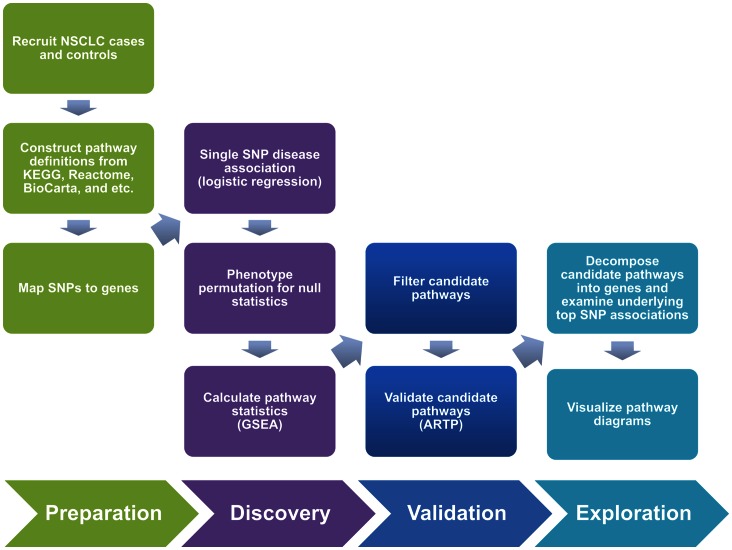
Overview of the Study.

In the present study, we used the GSEA-based pathway analysis suggested by Wang et al. [24] with our previous Korean lung cancer GWAS data, from 869 cases and 1,533 controls, with the hope of finding additional susceptibility loci and of obtaining insights into the underlying pathogenesis (**Figure 1**). Pathways showing high statistical significance were validated using another pathway-based method called adaptive rank truncated product (ARTP) [31]. In contrast to GSEA, ARTP is a self-contained test [32] that directly associates genes in a pathway to diseases and works independently of genes outside the pathway. We report seven pathways categorized into four cellular processes that showed consistent associations with Korean NSCLC susceptibility.

## Materials and Methods

### Ethics Statement

The study was approved by the Institutional Review Board and the Ethics Committee of National Cancer Center Korea. Blood samples were collected from NSCLC patients who visited National Cancer Center Korea and took a voluntary health questionnaire survey between May 2002 and December 2005. For each blood sample, written informed consent, approved by the Institutional Review Board members, was obtained. All clinical investigations were conducted according to the principles of the Declaration of Helsinki.

### Study Population

Initially, we recruited 2,441 Korean NSCLC cases and controls (871 cases and 1,570 controls) for this study. Most NSCLC samples (621 cases) were shared from a previous GWA study at National Cancer Center Korea [11], and additional 250 patients with NSCLC were newly recruited for genotyping. For comparison, genotyping data of 1,570 control subjects without cancer were provided by the Korean Association Resource (KARE) project, an ongoing population-based cohort study that has been conducted by Korea’s National Institute of Health (KNIH) and Center for Disease Control and Prevention (KCDC) since 2007 [33]. We applied strict sample quality control (QC) criteria, considering raw signal intensity, genotyping rate (≥95%), clinical information, and population stratification to filter unqualified samples. Specifically, we removed 19 samples with low-genotyping quality, 18 with insufficient demographic information, and two with gender misidentification. As a result, 869 cases and 1,533 controls passed the QC and remained for the subsequent analysis. Among 869 histologically confirmed NSCLC cases, 623 cases were adenocarcinomas, more than 70% of our NSCLC patients, 175 cases were squamous-cell carcinomas, and the rest were other NSCLC histological types (**Table 1**). More than 97% of subjects (n = 2,334) were genotyped using Affymetrix Genome-Wide Human SNP Array 5.0 (Affymetrix, Santa Clara, CA, USA), and the rest (n = 68) were genotyped using Affymetrix GeneChip Human Mapping 500 K Array Set. Upon genotyping and merging the data sets, we applied the following SNP QC filters: SNPs with a minor allele frequency (MAF) of less than 5% and genotyping call rates of less than 95%; Hardy-Weinberg equilibrium (HWE) test P-values≤0.0001 were excluded from further analysis.

**Table 1:** Demographic Features of Study Population.

				Multivariate		Multivariate (Stepwise)		Univariate		
Category	Subcategory	Cases (%)	Controls (%)	OR (95% CI)	P-value	OR (95% CI)	P-value	OR (95% CI)	P-value	
Histology	Adenocarcinoma	623 (71.7)								
	Squamous-Cell Carcinoma	175 (20.1)								
	Other NSCLC	71 (8.1)								
Gender	Male	466 (53.6)	892 (58.2)	0.48 (0.36–0.63)	<0.0001	0.48 (0.36–0.63)	0.0023	0.83 (0.70–0.98)	0.0303	
	Female	403 (46.4)	641 (41.8)							
Age	Median	60	59	0.98 (0.98–0.99)	0.0009	0.98 (0.98–0.99)	0.0104	0.99 (0.98–1.00)	0.0023	
	Range	25–85	40–70							
Smoking Status	Never-smoker	429 (49.4)	803 (52.4)	0.52 (0.39–0.68)	<0.0001	0.52 (0.39–0.68)	<0.0001	0.89 (0.75–1.05)	0.1557	
	Ever-smoker	440 (50.6)	730 (47.6)							

### Pathway Definition

We constructed a list of pathway definitions based on three major publicly available pathway databases: specifically, 217 gene sets from BioCarta [34], 186 from Kyoto Encyclopedia of Genes and Genomes (KEGG) [35], and 430 from Reactome [36]. We also included several curated pathway gene sets from SABiosciences [37], Sigma-Aldrich [38], Signal Transduction Knowledge Environment of Science magazine [39], and Signaling Pathway Database (SPAD) of Kyushu University [40] to cover a total of 880 biological pathways (**Appendix S1**).

To measure the performance of our analysis and to set a baseline, we also compiled six custom pathways from previously reported lung cancer susceptibility genes. First, we borrowed nine oncogenes (IL1B, MTHFR, AKAP9, CAMKK1, SEZ6L, FAS, FASLG, TP53, and TP53BP1) from a study conducted by the International Lung Cancer Consortium (ILCCO) [41]. According to the ILCCO study, genetic variants with strong evidence of an association with lung cancer risk belonged to genes from various cancer-related pathways, such as inflammation (IL1B), folate metabolism (MTHFR), regulatory function (AKAP9 and CAMKK1), cell adhesion (SEZL6), and apoptosis (FAS, FASL, TP53, TP53BP1, and BAT3). In addition, we adopted 11 NSCLC driver mutation genes (EGFR, KRAS, ERBB2, ALK, BRAF, PIK3CA, AKT1, MAP2K1, MET, ROS1, and NRAS) from a review by Pao and colleagues [42], [43]. In light of their review on how these genes affect cancer cell proliferation and survival, we included these clinically important genes as a basis for our positive controls. Finally, we added genes covered in lung cancer susceptibility loci reported in several previous GWA studies: C3ORF21 and TP63 from 3q28-29 [11], [44], TERT and CLPTM1L from 5p15 [5], [7], BAT and MSH5 from 6p21 [7], CHRNA5, CHRNA4, and CHRNA3 from 15q25 [8]–[10], and DNA repair genes (XRCC1, RRM1, ERCC1) [45]. We categorized genes by location or by function and designed six different combinations of lung cancer-related gene sets as positive controls (**Table 2**).

**Table 2:** Summary of Positive Control Tests.

		GSEA						ARTP	
		Additive			Dominant			Additive	Dominant
Gene Set	# of Genes	NES	NominalP-value	FDR	NES	NominalP-value	FDR	P-value	P-value
Master^1^	29	2.03	**0.024**	**0.064**	1.423	0.086	**0.169**	**0.001**	**0.001**
Without 3q28-29 Genes^2^	27	1.682	0.054	**0.084**	0.951	0.16	**0.248**	**0.007**	**0.004**
Without 5p15 Genes^3^	27	1.358	0.1	**0.097**	0.895	0.168	**0.211**	0.033	0.035
Without 6p21 Genes^4^	27	2.268	**0.014**	**0.088**	1.57	**0.07**	**0.194**	**0.004**	**0.002**
Without 15q25 Genes^5^	26	1.623	0.064	**0.073**	0.201	0.42	0.408	0.030	0.018
Without DNA Repair Genes^6^	27	2.231	**0.022**	**0.055**	1.675	0.048	0.334	**0.002**	**0.003**

1 IL1B, MTHFR, AKAP9, CAMKK1, SEZ6L, FAS, FASLG, TP53, TP53BP1, EGFR, KRAS, ERBB2, ALK, BRAF, PIK3CA, AKT1, MAP2K1, MET, ROS1, NRAS, C3ORF21, TP63, TERT, CLPTM1L, BAT3, MSH5, CHRNA3, CHRNA4, CHRNA5, XRCC1, RRM1, ERCC1.

2 3q28-29 Genes: C3ORF21, TP63.

3 5p15 Genes: TERT, CLPTM1L.

4 6p21 Genes: BAT3, MSH5.

5 15q25 Genes: CHRNA3, CHRNA4, CHRNA5.

6 DNA Repair Genes: XRCC1, RRM1, ERCC1.

* GSEA P-values≤0.025 and FDRs≤0.25, ARTP P-values≤0.01 are marked in bold.

### SNP Association and Mapping

Multivariate logistic regression of additive and dominant genetic models were fitted against disease status, with adjustments for age, gender, and smoking status, to accurately capture SNP association signals. Because we were interested primarily in the genetic effects of having a variant allele and MAF was generally low for our study pool, we excluded the recessive genetic model from our analysis.

Also, 20 kbp upstream of the 5′-end and 20 kbp downstream of the 3′-end were considered to be part of a gene, so as to include the coding region and regulatory elements. The most significantly associated SNP within a gene region was chosen to represent the whole gene area. Only gene sets with a minimum of 20 genes and a maximum of 200 genes were considered in the subsequent analysis.

### Pathway Analysis

We divided the pathway analysis into two-step screening and validation processes. First, we used an SNP adaptation of the GSEA method developed by Wang et al. [24] to screen candidate pathways associated with NSCLC risk. Then, using the ARTP algorithm developed by Yu et al. [31], we confirmed the statistical significances of candidate pathways.

#### Gene Set Enrichment Analysis (GSEA)

For each gene, the SNP with the highest test statistic (coefficient t-statistic from logistic regression in our case) was assigned to represent the gene. For all N genes, association statistics were sorted from largest to smallest (r_1_, r_2_,…,r_N_), and a weighted Kolmogorov-Smirnov-like (KS) running sum enrichment score (ES) of pathway S with N_P_ genes was calculated from the ranked list of genes [24].
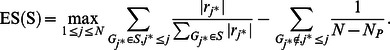



Because the calculation of ES(S) was based on a maximum statistic, pathways with large numbers of genes had definite advantages over smaller pathways. To compare statistics between pathways of different size, a normalized enrichment score (NES) was calculated as follows:
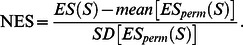



Finally, because we were testing multiple hypotheses at once, we assessed the expected false positive ratio by calculating the false discovery rate (FDR).
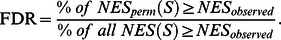



For our GSEA, 1,000 phenotype permutations were used to estimate each gene’s statistical significance.

#### Adaptive Rank Truncated Product (ARTP)

For all L genes in a pathway, the best SNP P-value of each gene was sorted from lowest to highest (P_1_, P_2_,…, P_L_), and the product of K smallest P-values in a pathway was calculated as follows:
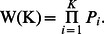



Normally, the truncation point K must be determined prior to using the RTP statistic. However, the ARTP method suggested by Yu et al. [31], which combines statistics derived from the observed dataset, makes it feasible to estimate the adjusted P-value independently of the size of the pathway. For our ARTP analysis, the same 1,000 permutations were used to assess the significance of each candidate pathway.

### Pathway Overlap Analysis

To compare similarities between pathways, the fractions of overlap between pathways were calculated, using the following equation:

Suppose A and B are pathways, then:
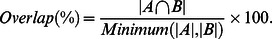



We used this equation to handle a situation where one pathway is a subset of the other. Thus, 100% similarity indicates that one pathway is a subset of the other, unless the two pathways are the same.

## Results

### Demographic Characteristics

The demographic features of the study population are shown in **Table 1**. In univariate analyses, gender and age showed statistical significance (P<0.05), while smoking status did not (P = 0.16). However, all three demographic features (gender, age, and smoking status) showed statistically significant associations (P<0.001) in multivariate analyses. Thus, we included all three as adjusting covariables during the logistic regression association analysis. Adenocarcinoma was the predominant histological type, representing more than 70% of our NSCLC samples, a composition consistent with the general Korean NSCLC population profile [4].

### Genotyping and Mapping

After applying the SNP QC criteria, including MAF and HWE, 440,530 genotyped SNPs were filtered down to 300,410 SNPs. From them, we removed SNPs residing in gene deserts and the remaining 147,970 SNPs were successfully mapped to our definition of genes (14,089 genes). Because we mapped SNPs located within 20 kbp upstream and downstream of a coding region as a gene, some SNPs located between genes were counted twice.

### Positive Control Tests

As shown in **Table 2**, our positive control test results showed a range of nominal P-values, from 0.014 to 0.42, and a range of FDRs, from 0.055 to 0.408, for the additive and dominant models in the GSEA method. For the ARTP method, P-values were stronger and ranged from 0.001 to 0.035 for the additive and dominant models. Thus, from the positive control tests, we used the following filtering criteria for the initial pathway discovery step using the GSEA method: P-values≤0.025 and FDRs≤25% in the additive or dominant genetic model were selected for subsequent analysis. For the ARTP method, P-values≤0.01 were considered noteworthy.

### Pathway Analysis

Among 880 pathways that we examined using the GSEA method, 11 passed the positive control filtering criteria (**Table 3**). The 11 candidate pathways were examined further using the ARTP method and their P-values were≤0.01 in the additive or dominant model, again confirming that these pathways had statistically significant associations with NSCLC, versus our positive controls. However, for the *Activation of the Pre-replicative Complex*, we found that the highly significant P-value of the additive model in the GSEA method differed from an insignificant P-value in the additive model in the ARTP method, so we excluded it from the subsequent analysis. Examining candidate pathways in depth following the results of the similarity tests, as shown in **Figure S1**, we found several pathways that resembled each other. For example, the *G1/S Transition* pathway was a subset of the *Cell Cycle* pathway, and when an inclusive relationship was found, we eliminated the superset, which, in this case, was the *Cell Cycle* pathway. Similarly, the *NRAGE Signals Death through JNK* pathway was a subset of the *Cell Death Signaling via NRAGE, NRIF, and NADE* pathway, which were subsets of the *P75 NTR Receptor Mediated Signaling* pathway. Thus, we removed two supersets of the *NRAGE Signals Death through JNK* pathway. The remaining seven candidate pathways were categorized into four types of cellular processes: membrane transport (*ABC Transporters*), intracellular signaling (*VEGF Signaling Pathway*, *Inositol Phosphate Metabolism*, *Phosphatidylinositol Signaling System*), cell cycle (*G1/S Check Point*, *G1/S Transition*), and programmed cell death (*NRAGE Signals Death through JNK*). Notably, the *G1/S Check Point* pathway had the smallest number of genes (25 genes), while maintaining statistical significance in both the GSEA and ARTP methods (dominant model). Using GSEA statistics as primary and ARTP statistics as secondary measurements, the *ABC Transporters* gene set showed the best NSCLC risk association among the seven final candidates with a GSEA nominal P-value<0.001, FDR = 0.122, and ARTP P-value = 0.001 (both dominant model). The second best was the *VEGF Signaling Pathway* with a GSEA nominal P-value<0.001, FDR = 0.107, and ARTP P-value = 0.008 (both dominant model).

**Table 3:** Candidate Pathways with P-value≤0.025 and FDR≤0.25.

Pathway	Source	# of Genes	GSEA						ARTP	
			Additive			Dominant			Additive	Dominant
			NES	Nominal P-value	FDR	NES	Nominal P-value	FDR	P-value	P-value
G1/S Transition	Reactome	77	3.139	**<0.001**	**0.203**	1.661	**0.042**	0.389	**0.003**	**0.005**
Activation of the Pre-replicative Complex	Reactome	21	2.940	**<0.001**	**0.223**	1.498	**0.030**	0.430	0.066	**0.010**
Cell Cycle	KEGG	99	2.764	**0.004**	0.286	2.935	**0.004**	**0.112**	**0.003**	**0.005**
G1/S Check Point	BioCarta	25	2.550	**0.010**	0.317	2.427	**0.004**	**0.211**	**0.006**	**0.013**
ABC Transporters	KEGG	38	2.295	**0.014**	0.362	2.848	**<0.001**	**0.122**	**0.004**	**0.001**
VEGF Signaling Pathway	KEGG	63	2.048	**0.016**	0.349	3.122	**<0.001**	**0.107**	**0.010**	**0.008**
Phosphatidylinositol Signaling System	KEGG	63	2.262	**0.016**	0.342	2.983	**0.002**	**0.114**	**0.002**	**0.007**
Inositol Phosphate Metabolism	KEGG	43	2.277	**0.024**	0.346	2.833	**0.002**	**0.120**	**0.009**	**0.001**
NRAGE Signals Death through JNK	Reactome	40	1.806	**0.032**	0.435	2.664	**0.006**	**0.144**	**0.003**	**0.001**
Cell Death Signaling via NRAGE, NRIF, and NADE	Reactome	49	1.497	0.058	0.448	2.590	**0.006**	**0.166**	**0.003**	**0.001**
P75 NTR Receptor Mediated Signaling	Reactome	62	1.409	0.084	0.469	2.525	**0.006**	**0.179**	**0.005**	**0.003**

* GSEA P-values≤0.025 and FDRs≤0.25, ARTP P-values≤0.01 are marked in bold.

For notable pathways in each cellular process category, SNP associations within gene sets were further examined (**Table 4** **, S1, S2, and S3**). Apart from the *ABC Transporters*, which was merely a collection of transmembrane protein pumps, network diagrams of notable pathways were drawn as shown in **Figures S2, S3, and S4**. The additional results for the pathway analysis in each subset according to the histologic types of lung cancer are described in **Appendix S2**.

**Table 4:** SNP Associations of Genes in “*ABC Transporters*.”

		Additive Model		Dominant Model				Additive Model		Dominant Model	
Gene	# of SNPs	Top SNP	P-value	Top SNP	P-value	Gene	# of SNPs	Top SNP	P-value	Top SNP	P-value
ABCA1	40	rs3905000	1.67×10^−3^	rs2066882	7.73×10^−3^	ABCC2	6	rs4148389	6.38×10^−2^	rs3740065	7.80×10^−2^
ABCA2	1	rs2049040	3.91×10^−1^	rs2049040	4.62×10^−1^	ABCC3	8	rs739922	3.16×10^−1^	rs739922	2.49×10^−1^
ABCA3	4	rs2014467	5.01×10^−2^	rs2014467	2.92×10^−2^	**ABCC4**	**64**	**rs9524822**	**6.90×10^−6^**	**rs9524822**	**1.73×10^−6^**
**ABCA4**	**37**	**rs4147868**	**2.37×10^−15^**	**rs4147868**	**7.86×10^−17^**	ABCC5	15	rs17750520	9.97×10^−3^	rs17750520	1.25×10^−2^
ABCA5	6	rs817126	1.92×10^−1^	rs817126	7.76×10^−2^	ABCC6	7	rs2283508	1.83×10^−1^	rs4780599	8.03×10^−2^
ABCA6	8	rs8081118	3.87×10^−3^	rs8081118	1.44×10^−3^	ABCC8	15	rs2077654	7.34×10^−2^	rs2077654	6.36×10^−2^
ABCA8	14	rs4147983	5.58×10^−3^	rs4147983	9.39×10^−3^	ABCC9	25	rs4148663	6.86×10^−2^	rs4148663	7.96×10^−2^
ABCA9	6	rs11077858	3.41×10^−1^	rs7215642	5.08×10^−1^	ABCC10	9	rs6907066	1.94×10^−1^	rs6907066	3.07×10^−1^
ABCA10	16	rs7217887	2.10×10^−1^	rs1024598	3.84×10^−1^	ABCD2	3	rs11172502	5.11×10^−1^	rs11172502	4.29×10^−1^
ABCA12	20	rs17430358	2.77×10^−2^	rs17430358	1.36×10^−2^	ABCD3	4	rs1749541	6.59×10^−1^	rs4148057	8.64×10^−1^
ABCA13	56	rs10236551	2.66×10^−3^	rs10236551	1.56×10^−3^	ABCD4	3	rs2074946	8.85×10^−1^	rs4148077	6.85×10^−1^
ABCB1	19	rs2235047	1.59×10^−1^	rs12670317	1.99×10^−1^	ABCG1	18	rs3787968	2.52×10^−2^	rs170444	1.04×10^−2^
ABCB4	12	rs31659	1.11×10^−2^	rs2097937	2.21×10^−2^	ABCG2	11	rs3114015	2.58×10^−1^	rs1481014	2.10×10^−1^
ABCB5	18	rs17143334	1.06×10^−1^	rs10488577	1.43×10^−1^	ABCG4	1	rs674424	7.46×10^−1^	rs674424	8.16×10^−1^
ABCB8	2	rs2303922	3.11×10^−1^	rs2303922	2.60×10^−1^	ABCG5	4	rs2278357	3.61×10^−2^	rs10439467	6.18×10^−2^
ABCB9	2	rs4275659	3.81×10^−1^	rs4275659	3.04×10^−1^	ABCG8	3	rs4148202	6.57×10^−1^	rs4148202	6.63×10^−1^
ABCB10	1	rs10916508	3.40×10^−1^	rs10916508	3.69×10^−1^	CFTR	13	rs4148689	3.12×10^−2^	rs4148689	2.46×10^−2^
ABCB11	22	rs6759156	1.59×10^−2^	rs6759156	7.65×10^−2^	TAP1	2	rs12529313	8.24×10^−1^	rs12529313	6.83×10^−1^
**ABCC1**	**33**	**rs12921623**	**2.07×10^−7^**	**rs12921623**	**3.33×10^−11^**	TAP2	6	rs241429	1.66×10^−1^	rs241429	1.34×10^−1^

* P-values<5×10^−4^ was considered genome-wide level significant and marked in bold.

### Comparison with Other Lung Cancer Study

In a differentially expressed gene study with normal lung tissue, conducted by Falvella et al. [46], a gene-expression signature consisting of 85 genes was suggested to distinguish lung adenocarcinoma patients from other cancer patients. We adopted 67 genes that were available in NCBI human genome build 36 and applied the same pathway analysis procedure. Using our genotyping data, SNP associations were successfully mapped to 54 genes. With the gene set including these 54 genes, the GSEA method showed nominal P-values of <0.001 for both additive and dominant models and FDR values of 2.6% and 11.7% for the additive and dominant models, respectively. Similarly, the ARTP method yielded P-values of 9.99×10^−4^ and 3.00×10^−3^ for the additive and dominant models for this gene set, respectively. This result again confirmed that our pathway-based analysis was legitimate and consistent with other lung cancer study involving mRNA-based gene-expression analysis. The results from association analysis of SNPs in 54 genes were shown in **Table S4**.

## Discussion

Recent advances in high-throughput SNP genotyping technology have generated massive amounts of genotyping data and have led to valuable results regarding common genetic variants associated with various diseases through GWASs. However, the reports have focused mainly on a small portion of associations that qualify for genome-wide significance level and most associations, with moderate statistical powers, are hard to interpret. Using prior biological knowledge, pathway-based association approaches have recently opened up a new way to examine associations between GWAS results and complex molecular networks. Pathway-based analysis incorporates association data for functionally related genes and translates them into disease susceptibility information.

In this study, we used a mix of contrasting pathway analyses using Korean lung cancer GWAS data, consisting of 869 NSCLC cases and 1,533 controls. We previously reported significant associations between polymorphisms at chromosomes 3q28, 3q29, and 5p15 and Korean lung cancer susceptibility [11], [44]. We first used a GSEA-based method with genome-wide SNP array data to preliminarily screen for candidate pathways associated with lung cancer susceptibility. Among 11 candidate pathways, we selected 7 that were also confirmed by the ARTP method. Four cellular signaling pathways, *VEGF Signaling Pathway*, *G1/S Check Point*, *NRAGE Signals Death through JNK*, and *ABC Transporters,* were highly enriched with signals associated with lung cancer risk. Unlike GWASs, P-values for SNPs associated with genes were generally moderately significant and only a few of them would qualify at the so called genome-wide significance level. This indicated that pathway statistics emphasized the effects of subtle, but systematic, patterns within a gene set instead of a few peak associations within a gene set.

Gene members of the ATP-binding cassette (ABC) transporter family have essential functions in transporting various substrates, such as ions, sugars, lipids, and proteins, under physiological conditions. For many years, researchers have discovered that the *ABC Transporters* play significant roles in cancer chemotherapy and are responsible for multidrug resistance (MDR), in the form of an ATP-driven drug pump [47]. It is believed that overexpression of the *ABC Transporters* reduces intracellular drug levels via enhanced efflux of chemotherapeutic agents, resulting in drug insensitivity, which can lead to cancer chemotherapy failure [48]. On the other hand, *ABC Transporters* can also function as effective carcinogen exporters, keeping cells free of harmful chemicals and carcinogens. Prolonged impairments or changes in gene expression of these transmembrane protein pumps can increase potential cancer risk. Tobacco-specific nitrosamine 4-(methylnitrosamino)-1-(3-pyridyl)-1-butanone (NNK) is one of the most potent carcinogens of cigarette smoke that has been shown to cause lung cancer in rodents [49], [50]. When these toxins are inhaled, the *ABC Transporters* encoded by ABCB1 and ABCC1 effectively eliminate these carcinogens from the lungs. Researchers have discovered that common polymorphisms of ABCB1 and ABCC1 can influence metabolism and disposition of the well-established carcinogen, NNK, and potentially increase the lung cancer risk [51]. As shown in **Table 4**, ABCA4, ABCC1, and ABCC showed significant associations with lung cancer susceptibility (P-values<0.0005). In our subsequent subgroup analysis, ever-smokers showed a more significant association than never-smokers, as shown in **Table S5**. Thus, it is possible to modulate individual lung cancer risk according to genetic polymorphisms in those genes through different cellular functions.

The *VEGF Signaling Pathway* also ranked high in our pathway analysis. Members of this pathway may influence an angiogenesis-dependent biological pathway, which is a critical component of oncogenesis. Associations between genetic polymorphisms in VEGF/VEGFR and the risk of developing cancers have been reported in various cancer types, including lung cancer [52]. Among 63 gene members, phosphatidylinositol 3-kinase (PIK3R5), phospholipase C (PLCG2), and SHC adaptor protein showed strong associations with lung cancer susceptibility in our GWAS (**Table S1, Figure S2**).

Although a pathway-based approach is an attractive trial in a post-GWAS era, we note some limitations in our design. In our study, following the SNP QC, associations of more than 300,000 SNPs remained and were analyzed, but only about half of them were mapped successfully, to 14,089 genes. Many SNPs within non-coding regions were simply neglected. Unfortunately, with the current GWAS genotyping platform, the number of markers that cover genes is limited. Moreover, even the 880 pathways we analyzed were clearly an incomplete set of pathways because many human genes have not yet been assigned to pathways because their function(s) are unknown. Finally, our GWAS set containing genome-wide SNP data of more than 2,400 Koreans originated from a single population. The aim of the study was to find pathways associated with lung cancer susceptibility within Korean population, and we could not find a comparable Korean lung cancer GWAS data set for validating our findings. As more data become available, our results should be compared to other East Asian populations considering ethnic differences.

In conclusion, we demonstrated that lung cancer susceptibility can be linked to biological pathways using GWAS data, and multiple subtle association signals can be interpreted in a systematic manner. Our results suggest that genetic variation in genes involved in four signaling pathways may contribute to individual lung cancer susceptibility. Moreover, our findings indicate that pathway-based approaches are important analytical methods in a post-GWAS era that could possibly be used to address the functional relevance of genetic susceptibility.

## Supporting Information

Figure S1 **Similarities between Candidate Pathways. (Unit: %).**
(TIF)

Figure S2 **Pathway Diagram of “VEGF Signaling”.**
(TIF)

Figure S3 **Pathway Diagram of “G1/S Check Point”.**
(TIF)

Figure S4 **Pathway Diagram of “NRAGE Signals Death through JNK”.**
(TIF)

Table S1 SNP Associations of Genes in “VEGF Signaling Pathway”.
(DOC)

Table S2 SNP Associations of Genes in “G1/S Check Point”.
(DOC)

Table S3 SNP Associations of Genes in “NRAGE Signals Death through JNK”.
(DOC)

Table S4 SNP Associations of 54 Genes in “Smokers with Lung Cancer” Gene Expression Study by Falvella et al.
(DOC)

Table S5 Comparison of “ABC Transporters” Pathway Genes Between Never-smoker and Ever-smoker.
(DOC)

Appendix S1 **List of 880 Pathways.**
(XLSX)

Appendix S2 **Pathway Analysis of 2156 Adenocarcinoma Only Group and 1806 Squamous-Cell Carcinoma Only Group.**
(XLSX)
