# Supplementary figures and images for: Pathway-Based Analysis Using Genome-wide Association Data from a Korean Non-Small Cell Lung Cancer Study
Source: PLoS One. 2013 Jun 6;8(6):e65396. doi: 10.1371/journal.pone.0065396 (PMC3675130; doi:10.1371/journal.pone.0065396)

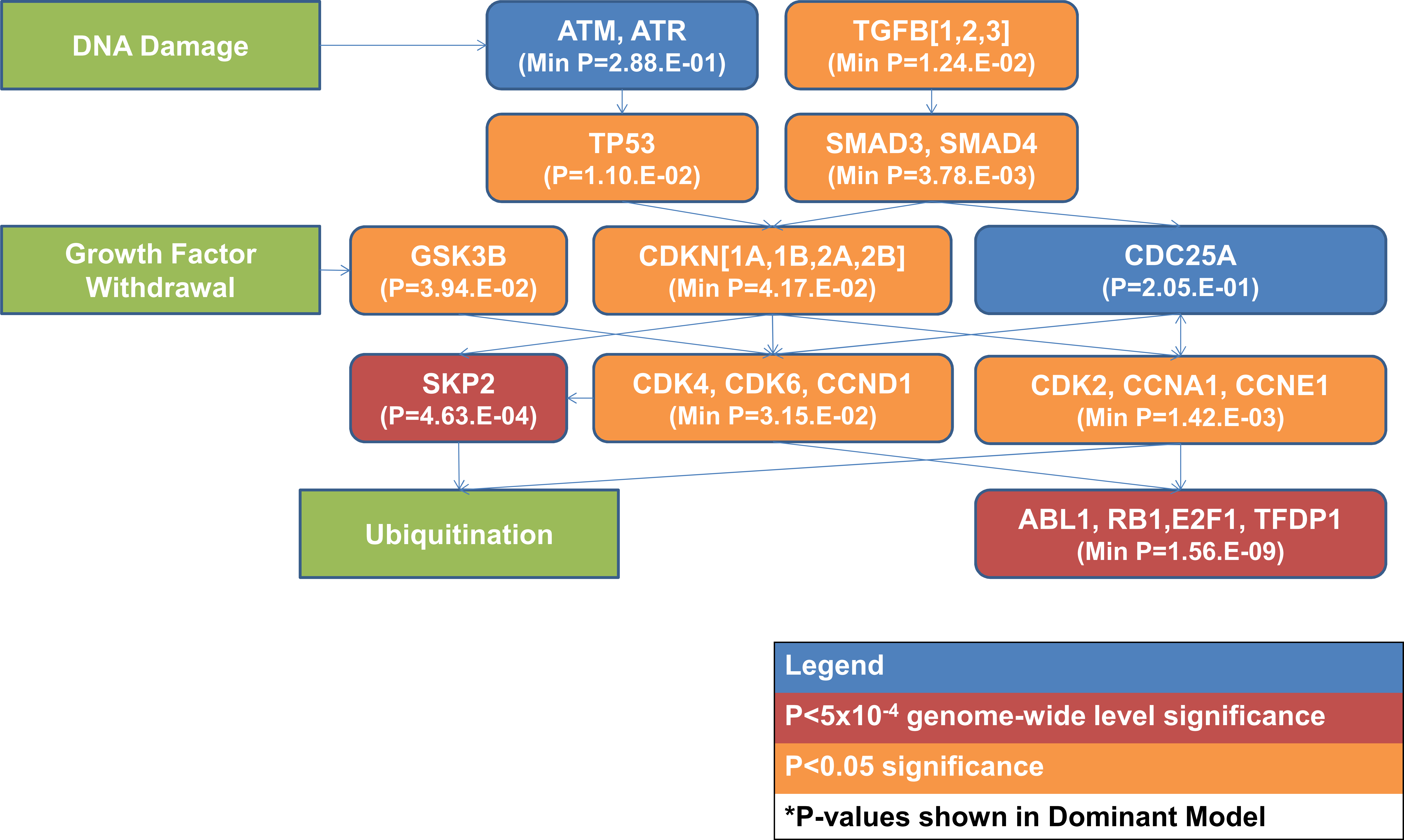

Supplement: Figure S3 — Pathway Diagram of “G1/S Check Point”. (TIF) [file pone.0065396.s003.tif]

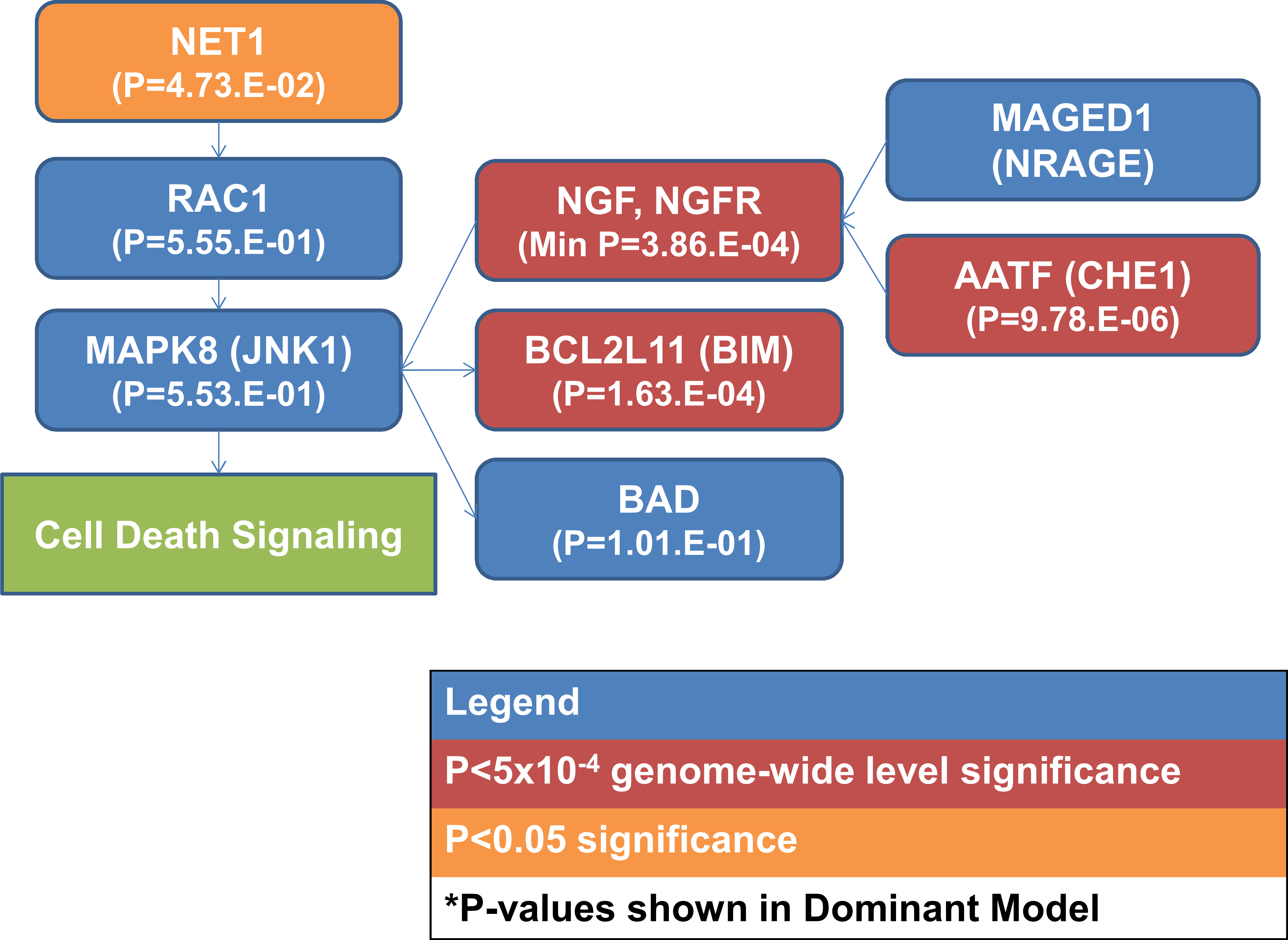

Supplement: Figure S4 — Pathway Diagram of “NRAGE Signals Death through JNK”. (TIF) [file pone.0065396.s004.tif]
