# Supplementary material for: Pathway-Based Analysis Using Genome-wide Association Data from a Korean Non-Small Cell Lung Cancer Study
Source: PLoS One. 2013 Jun 6;8(6):e65396. doi: 10.1371/journal.pone.0065396 (PMC3675130; doi:10.1371/journal.pone.0065396)
Supplement: Table S1 — SNP Associations of Genes in “VEGF Signaling Pathway”. (DOC) [file pone.0065396.s005.doc]

**Table S1. SNP Associations of Genes in “VEGF Signaling Pathway.**”

|  |  |  | **Additive Model** | |  | **Dominant Model** | |  |  |  |  | **Additive Model** | |  | **Dominant Model** | |
| --- | --- | --- | --- | --- | --- | --- | --- | --- | --- | --- | --- | --- | --- | --- | --- | --- |
| **Gene** | **# of SNPs** |  | **Top SNP** | **P-value** |  | **Top SNP** | **P-value** |  | **Gene** | **# of SNPs** |  | **Top SNP** | **P-value** |  | **Top SNP** | **P-value** |
| AKT1 | 2 |  | rs1130214 | 4.74.E-01 |  | rs2494731 | 7.95.E-02 |  | PLA2G1B | 4 |  | rs2522136 | 5.45.E-02 |  | rs2522136 | 8.63.E-02 |
| AKT2 | 2 |  | rs7250897 | 2.43.E-01 |  | rs7250897 | 3.43.E-01 |  | PLA2G2A | 7 |  | rs876018 | 2.57.E-01 |  | rs876018 | 3.18.E-01 |
| AKT3 | 17 |  | rs2881274 | 1.02.E-01 |  | rs2881274 | 3.85.E-02 |  | PLA2G2C | 4 |  | rs6667486 | 1.79.E-01 |  | rs6667486 | 5.09.E-01 |
| BAD | 1 |  | rs11231741 | 1.36.E-01 |  | rs11231741 | 1.01.E-01 |  | PLA2G2D | 4 |  | rs636584 | 1.32.E-01 |  | rs636584 | 1.60.E-01 |
| CASP9 | 6 |  | rs2042370 | 5.29.E-02 |  | rs4646044 | 7.24.E-03 |  | PLA2G2E | 5 |  | rs41416448 | 1.94.E-01 |  | rs41416448 | 1.74.E-01 |
| CDC42 | 8 |  | rs2268177 | 1.86.E-01 |  | rs2473277 | 4.19.E-01 |  | PLA2G2F | 1 |  | rs4654839 | 2.27.E-02 |  | rs4654839 | 1.22.E-02 |
| CHP | 3 |  | rs16971735 | 2.68.E-01 |  | rs16971735 | 2.46.E-01 |  | PLA2G3 | 4 |  | rs3761430 | 7.46.E-02 |  | rs3761430 | 5.50.E-03 |
| CHP2 | 3 |  | rs194802 | 8.79.E-01 |  | rs9935512 | 6.37.E-01 |  | PLA2G4A | 18 |  | rs17591814 | 8.15.E-03 |  | rs17591814 | 3.83.E-03 |
| HSPB1 | 1 |  | rs2868371 | 6.41.E-01 |  | rs2868371 | 3.11.E-01 |  | JMJD7-PLA2G4B | 4 |  | rs1206842 | 4.17.E-01 |  | rs2412640 | 4.38.E-01 |
| KDR | 6 |  | rs17085310 | 8.75.E-02 |  | rs2219471 | 4.23.E-02 |  | PLA2G4E | 6 |  | rs1628549 | 1.06.E-03 |  | rs1628549 | 2.28.E-03 |
| KRAS | 12 |  | rs6487465 | 2.19.E-01 |  | rs4623993 | 2.09.E-01 |  | PLA2G5 | 9 |  | rs656110 | 8.46.E-02 |  | rs656110 | 2.61.E-02 |
| MAP2K1 | 6 |  | rs16953563 | 2.11.E-02 |  | rs4255740 | 6.52.E-03 |  | PLA2G6 | 6 |  | rs4821754 | 6.89.E-02 |  | rs4821754 | 2.15.E-02 |
| MAPK1 | 12 |  | rs5999749 | 9.05.E-03 |  | rs5999749 | 5.27.E-02 |  | PLA2G12A | 1 |  | rs1541373 | 1.40.E-01 |  | rs1541373 | 8.08.E-01 |
| MAPK13 | 3 |  | rs2071864 | 9.15.E-02 |  | rs2859131 | 6.49.E-02 |  | PPP3CA | 48 |  | rs2850371 | 1.16.E-02 |  | rs2850359 | 5.97.E-02 |
| MAPK14 | 6 |  | rs3804452 | 2.01.E-01 |  | rs3804452 | 1.60.E-01 |  | PPP3CB | 3 |  | rs12644 | 9.85.E-02 |  | rs1041532 | 1.91.E-01 |
| MAPKAPK2 | 5 |  | rs11119385 | 1.84.E-01 |  | rs11119385 | 2.68.E-01 |  | PPP3CC | 13 |  | rs9785089 | 6.98.E-02 |  | rs9785089 | 1.30.E-01 |
| MAPKAPK3 | 9 |  | rs876104 | 2.06.E-02 |  | rs876104 | 3.28.E-01 |  | PPP3R1 | 9 |  | rs6546359 | 5.55.E-01 |  | rs930653 | 3.98.E-01 |
| NFAT5 | 13 |  | rs39999 | 4.34.E-01 |  | rs8045705 | 5.09.E-01 |  | PPP3R2 | 1 |  | rs1407877 | 1.06.E-01 |  | rs1407877 | 2.42.E-01 |
| NFATC1 | 6 |  | rs1660144 | 5.55.E-01 |  | rs1660144 | 2.59.E-01 |  | PRKCA | 90 |  | rs16960228 | 1.34.E-01 |  | rs16960228 | 2.17.E-02 |
| NFATC2 | 31 |  | rs6013210 | 3.92.E-03 |  | rs6123045 | 1.68.E-03 |  | PRKCB | 56 |  | rs2239339 | 4.77.E-03 |  | rs2239339 | 3.56.E-03 |
| NFATC3 | 2 |  | rs8056649 | 3.88.E-01 |  | rs8056649 | 6.58.E-01 |  | PTGS2 | 6 |  | rs2383529 | 9.33.E-03 |  | rs2383529 | 3.89.E-03 |
| NFATC4 | 3 |  | rs9788516 | 2.87.E-01 |  | rs9788516 | 2.08.E-01 |  | PTK2 | 37 |  | rs4961290 | 1.22.E-03 |  | rs4961290 | 3.54.E-03 |
| NOS3 | 1 |  | rs3918188 | 2.90.E-01 |  | rs3918188 | 2.24.E-01 |  | PXN | 3 |  | rs2283363 | 2.54.E-02 |  | rs2283363 | 2.57.E-02 |
| PIK3CA | 5 |  | rs1607237 | 2.67.E-01 |  | rs2677764 | 1.60.E-01 |  | RAC1 | 5 |  | rs702484 | 3.97.E-01 |  | rs836554 | 5.55.E-01 |
| PIK3CB | 1 |  | rs12493155 | 3.90.E-01 |  | rs12493155 | 9.04.E-01 |  | RAC2 | 2 |  | rs1476002 | 6.35.E-02 |  | rs1476002 | 1.20.E-01 |
| PIK3CD | 6 |  | rs4240896 | 1.06.E-02 |  | rs4240896 | 1.22.E-02 |  | RAF1 | 2 |  | rs2442809 | 2.89.E-01 |  | rs2442809 | 3.58.E-01 |
| PIK3CG | 11 |  | rs4730205 | 6.04.E-02 |  | rs4730205 | 6.98.E-02 |  | SH2D2A | 1 |  | rs2150906 | 2.31.E-01 |  | rs2150906 | 2.86.E-01 |
| PIK3R1 | 14 |  | rs706713 | 1.15.E-01 |  | rs706713 | 2.48.E-01 |  | **SHC2** | **3** |  | **rs2075019** | **7.96.E-06** |  | **rs2075019** | **7.78.E-08** |
| PIK3R3 | 7 |  | rs1613296 | 4.02.E-03 |  | rs1613296 | 1.38.E-02 |  | SPHK1 | 2 |  | rs9889656 | 1.19.E-02 |  | rs9889656 | 2.05.E-02 |
| **PIK3R5** | **4** |  | **rs12945251** | **2.30.E-06** |  | **rs12945251** | **6.69.E-07** |  | SRC | 3 |  | rs6018100 | 6.59.E-01 |  | rs16986606 | 6.59.E-01 |
| PLCG1 | 3 |  | rs3795131 | 6.30.E-01 |  | rs3795131 | 3.50.E-01 |  | VEGFA | 4 |  | rs943084 | 1.29.E-02 |  | rs4714696 | 1.018.E-02 |
| **PLCG2** | **33** |  | rs4405545 | 9.12.E-04 |  | **rs4405545** | **3.75.E-04** |  |  |  |  |  |  |  |  |  |
| ^*^ P-values < 5x10^-4^ was considered genome-wide level significant and marked in bold | | | | | | | | | | | | | | | | |
