# Supplementary material for: Pathway-Based Analysis Using Genome-wide Association Data from a Korean Non-Small Cell Lung Cancer Study
Source: PLoS One. 2013 Jun 6;8(6):e65396. doi: 10.1371/journal.pone.0065396 (PMC3675130; doi:10.1371/journal.pone.0065396)
Supplement: Table S2 — SNP Associations of Genes in “G1/S Check Point”. (DOC) [file pone.0065396.s006.doc]

**Table S2. SNP Associations of Genes in “G1/S Check Point.”**

|  |  |  | **Additive Model** | |  | **Dominant Model** | |
| --- | --- | --- | --- | --- | --- | --- | --- |
| **Gene** | **# of SNPs** |  | **Top SNP** | **P-value** |  | **Top SNP** | **P-value** |
| **ABL1** | **23** |  | **rs4740377** | **2.47.E-08** |  | **rs4740377** | **1.56.E-09** |
| ATM | 10 |  | rs624366 | 1.76.E-01 |  | rs11212570 | 2.88.E-01 |
| ATR | 4 |  | rs12639363 | 3.12.E-01 |  | rs12639363 | 5.08.E-01 |
| CCNA1 | 3 |  | rs10514840 | 2.97.E-01 |  | rs8002093 | 3.70.E-01 |
| CCND1 | 1 |  | rs654240 | 8.38.E-01 |  | rs654240 | 6.80.E-01 |
| CCNE1 | 7 |  | rs34331990 | 2.36.E-03 |  | rs34331990 | 1.42.E-03 |
| CDC25A | 5 |  | rs1380053 | 3.74.E-01 |  | rs6796490 | 2.05.E-01 |
| CDK2 | 1 |  | rs2069408 | 3.14.E-01 |  | rs2069408 | 2.81.E-01 |
| CDK4 | 1 |  | rs2069502 | 4.77.E-02 |  | rs2069502 | 4.15.E-02 |
| CDK6 | 24 |  | rs6964803 | 3.07.E-02 |  | rs2282978 | 3.15.E-02 |
| CDKN1A | 12 |  | rs762623 | 5.05.E-02 |  | rs762623 | 4.17.E-02 |
| CDKN1B | 6 |  | rs17758650 | 2.59.E-01 |  | rs10845615 | 2.42.E-01 |
| CDKN2A | 2 |  | rs717326 | 4.24.E-01 |  | rs717326 | 1.85.E-01 |
| CDKN2B | 6 |  | rs7044859 | 2.49.E-01 |  | rs643319 | 1.91.E-01 |
| E2F1 | 1 |  | rs3213183 | 2.61.E-02 |  | rs3213183 | 2.00.E-02 |
| GSK3B | 16 |  | rs334541 | 1.34.E-01 |  | rs4624596 | 3.94.E-02 |
| RB1 | 6 |  | rs198584 | 1.79.E-01 |  | rs2854342 | 3.26.E-01 |
| **SKP2** | **3** |  | **rs7731023** | **4.96.E-04** |  | **rs7731023** | **4.63.E-04** |
| SMAD3 | 24 |  | rs16950543 | 4.29.E-03 |  | rs16950543 | 3.78.E-03 |
| SMAD4 | 6 |  | rs10502913 | 4.20.E-01 |  | rs10502913 | 4.05.E-01 |
| TFDP1 | 11 |  | rs12428926 | 1.11.E-02 |  | rs12428926 | 2.40.E-02 |
| TGFB1 | 1 |  | rs2241716 | 9.25.E-02 |  | rs2241716 | 8.20.E-02 |
| TGFB2 | 16 |  | rs10482751 | 3.63.E-03 |  | rs10482751 | 1.24.E-02 |
| TGFB3 | 2 |  | rs3917148 | 4.66.E-02 |  | rs3917148 | 5.66.E-02 |
| TP53 | 2 |  | rs12602273 | 2.13.E-02 |  | rs12602273 | 1.10.E-02 |
| ^*^ P-values < 5x10^-4^ was considered genome-wide level significant and marked in bold | | | | | | | |
