# Supplementary material for: Pathway-Based Analysis Using Genome-wide Association Data from a Korean Non-Small Cell Lung Cancer Study
Source: PLoS One. 2013 Jun 6;8(6):e65396. doi: 10.1371/journal.pone.0065396 (PMC3675130; doi:10.1371/journal.pone.0065396)
Supplement: Table S3 — SNP Associations of Genes in “NRAGE Signals Death through JNK”. (DOC) [file pone.0065396.s007.doc]

**Table S3. SNP Associations of Genes in “NRAGE Signals Death through JNK.**”

|  |  |  | **Additive Model** | |  | **Dominant Model** | |  |  |  |  | **Additive Model** | |  | **Dominant Model** | |
| --- | --- | --- | --- | --- | --- | --- | --- | --- | --- | --- | --- | --- | --- | --- | --- | --- |
| **Gene** | **# of SNPs** |  | **Top SNP** | **P-value** |  | **Top SNP** | **P-value** |  | **Gene** | **# of SNPs** |  | **Top SNP** | **P-value** |  | **Top SNP** | **P-value** |
| **AATF (CHE1)** | **8** |  | **rs1564796** | **2.05.E-05** |  | **rs1564796** | **9.78.E-06** |  | MAPK8 (JNK1) | 5 |  | rs10857560 | 5.95.E-01 |  | rs10857560 | 5.53.E-01 |
| **ABR** | **10** |  | rs7207980 | 2.03.E-03 |  | **rs7207980** | **1.80.E-04** |  | MCF2L | 14 |  | rs7326792 | 6.34.E-02 |  | rs7326792 | 1.02.E-01 |
| **AKAP13** | **64** |  | **rs41388746** | **4.06.E-04** |  | rs41388746 | 2.10.E-03 |  | NET1 | 9 |  | rs12572605 | 6.38.E-02 |  | rs4881459 | 4.73.E-02 |
| ARHGEF2 (GEF) | 1 |  | rs2016251 | 7.05.E-01 |  | rs2016251 | 6.41.E-01 |  | **NGEF** | **22** |  | **rs12477794** | **2.07.E-04** |  | **rs12477794** | **1.70.E-04** |
| ARHGEF3 | 50 |  | rs2054857 | 4.25.E-03 |  | rs4499565 | 4.02.E-03 |  | **NGF** | **22** |  | rs2982742 | 2.81.E-03 |  | **rs2982742** | **3.86.E-04** |
| ARHGEF4 | 9 |  | rs12470817 | 1.11.E-01 |  | rs12470817 | 5.02.E-02 |  | NGFR | 5 |  | rs2584665 | 7.24.E-02 |  | rs10491195 | 1.58.E-02 |
| ARHGEF7 | 23 |  | rs7984371 | 3.07.E-02 |  | rs7984371 | 3.05.E-02 |  | OBSCN | 22 |  | rs10916293 | 3.51.E-02 |  | rs17640616 | 6.07.E-02 |
| ARHGEF11 | 15 |  | rs1007604 | 3.60.E-01 |  | rs1007604 | 3.27.E-01 |  | PLEKHG5 | 1 |  | rs4908901 | 2.74.E-01 |  | rs4908901 | 9.02.E-01 |
| ARHGEF12 | 11 |  | rs661139 | 1.76.E-01 |  | rs17123848 | 3.95.E-01 |  | PREX1 | 33 |  | rs6066838 | 2.10.E-02 |  | rs2426101 | 1.68.E-02 |
| ARHGEF16 | 1 |  | rs2487681 | 9.44.E-01 |  | rs2487681 | 6.17.E-01 |  | RAC1 | 5 |  | rs702484 | 3.97.E-01 |  | rs836554 | 5.55.E-01 |
| ARHGEF17 | 5 |  | rs7342205 | 3.03.E-01 |  | rs7342205 | 3.76.E-01 |  | RASGRF1 | 21 |  | rs11635204 | 5.31.E-02 |  | rs11635204 | 4.98.E-02 |
| ARHGEF18 | 6 |  | rs12979990 | 3.63.E-01 |  | rs12979990 | 3.12.E-01 |  | RASGRF2 | 48 |  | rs26893 | 1.23.E-02 |  | rs26893 | 3.99.E-03 |
| BAD | 1 |  | rs11231741 | 1.36.E-01 |  | rs11231741 | 1.01.E-01 |  | SOS1 | 10 |  | rs6758330 | 2.00.E-01 |  | rs297130 | 2.83.E-01 |
| **BCL2L11 (BIM)** | **5** |  | rs1837369 | 3.61.E-02 |  | **rs1837369** | **1.63.E-04** |  | SOS2 | 8 |  | rs1955926 | 9.36.E-02 |  | rs10483600 | 1.34.E-02 |
| ECT2 | 4 |  | rs11714381 | 2.67.E-01 |  | rs11714381 | 2.79.E-01 |  | TIAM1 | 68 |  | rs2268223 | 1.88.E-03 |  | rs16988223 | 1.11.E-02 |
| FGD2 | 7 |  | rs831512 | 1.67.E-02 |  | rs831512 | 1.01.E-02 |  | TIAM2 | 14 |  | rs931312 | 1.10.E-02 |  | rs2882936 | 3.38.E-02 |
| **FGD3** | **6** |  | **rs6479445** | **2.79.E-04** |  | **rs6479445** | **1.54.E-05** |  | TRIO | 31 |  | rs16903326 | 1.12.E-01 |  | rs12659261 | 7.40.E-02 |
| FGD4 | 19 |  | rs10506089 | 2.45.E-02 |  | rs10506089 | 3.01.E-02 |  | VAV1 | 6 |  | rs164016 | 2.99.E-02 |  | rs164016 | 2.78.E-01 |
| ITSN1 | 18 |  | rs2834252 | 1.88.E-01 |  | rs2834268 | 1.49.E-01 |  | **VAV2** | **25** |  | **rs2520006** | **3.45.E-04** |  | **rs12378050** | **1.61.E-05** |
| **KALRN** | **129** |  | **rs485887** | **4.31.E-09** |  | **rs485887** | **4.76.E-10** |  | VAV3 | 62 |  | rs3790676 | 6.14.E-02 |  | rs3790676 | 8.78.E-02 |
| ^*^ P-values < 5x10^-4^ was considered genome-wide level significant and marked in bold | | | | | | | | | | | | | | | | |
