# Supplementary material for: Pathway-Based Analysis Using Genome-wide Association Data from a Korean Non-Small Cell Lung Cancer Study
Source: PLoS One. 2013 Jun 6;8(6):e65396. doi: 10.1371/journal.pone.0065396 (PMC3675130; doi:10.1371/journal.pone.0065396)
Supplement: Table S4 — SNP Associations of 54 Genes in “Smokers with Lung Cancer” Gene Expression Study by Falvella et al. (DOC) [file pone.0065396.s008.doc]

**Table S4. SNP Associations of 54 Genes in “Smokers with Lung Cancer” Gene Expression S**tudy by Falvella et al.

|  |  |  |  | **Additive Model** | |  | **Dominant Model** | |
| --- | --- | --- | --- | --- | --- | --- | --- | --- |
| **Gene** | **Official Full Name** | **# of SNPs** |  | **Top SNP** | **P-value** |  | **Top SNP** | **P-value** |
| **ACTR2** | **ARP2 actin-related protein 2 homolog (yeast)** | **2** |  | **rs17030062** | **3.58.E-01** |  | **rs17030062** | **8.34.E-02** |
| **ADAMTS1** | **ADAM metallopeptidase with thrombospondin type 1 motif, 1** | **10** |  | **rs229037** | **3.25.E-05** |  | **rs229037** | **1.15.E-05** |
| AHRR | aryl-hydrocarbon receptor repressor | 8 |  | rs2672753 | 3.02.E-02 |  | rs2672753 | 3.28.E-02 |
| ANK2 | ankyrin 2, neuronal | 91 |  | rs41434448 | 1.58.E-03 |  | rs41434448 | 2.51.E-03 |
| APOL6 | apolipoprotein L, 6 | 2 |  | rs2103768 | 1.32.E-03 |  | rs2103768 | 2.01.E-03 |
| AQP4 | aquaporin 4 | 8 |  | rs335931 | 2.54.E-01 |  | rs335931 | 1.13.E-01 |
| ATP6V0D2 | ATPase, H+ transporting, lysosomal 38kDa, V0 subunit d2 | 15 |  | rs2466326 | 1.31.E-02 |  | rs2466326 | 4.92.E-02 |
| AZGP1 | alpha-2-glycoprotein 1, zinc | 3 |  | rs4215 | 3.09.E-01 |  | rs6465760 | 5.98.E-01 |
| BTG2 | BTG family, member 2 | 6 |  | rs2486942 | 2.48.E-01 |  | rs17534202 | 2.06.E-01 |
| CAMKK2 | calcium/calmodulin-dependent protein kinase kinase 2, beta | 6 |  | rs3794207 | 8.33.E-04 |  | rs3794207 | 3.89.E-03 |
| CCL20 | chemokine (C-C motif) ligand 20 | 4 |  | rs1911135 | 7.31.E-02 |  | rs1911135 | 1.04.E-01 |
| CTNNA1 | catenin (cadherin-associated protein), alpha 1, 102kDa | 15 |  | rs160404 | 2.07.E-03 |  | rs160404 | 4.67.E-03 |
| DUSP1 | dual specificity phosphatase 1 | 2 |  | rs17075176 | 7.21.E-02 |  | rs17075176 | 3.41.E-02 |
| EPAS1 | endothelial PAS domain protein 1 | 19 |  | rs7583392 | 4.48.E-03 |  | rs7583392 | 3.56.E-03 |
| **FER** | **fer (fps/fes related) tyrosine kinase** | **22** |  | **rs7731193** | **6.41.E-05** |  | **rs7731193** | **1.38.E-04** |
| FKBP5 | FK506 binding protein 5 | 9 |  | rs7757037 | 6.99.E-03 |  | rs755658 | 6.36.E-02 |
| FOLH1 | folate hydrolase (prostate-specific membrane antigen) 1 | 3 |  | rs16906173 | 1.65.E-01 |  | rs16906173 | 1.74.E-01 |
| FUS | fused in sarcoma | 1 |  | rs4889537 | 1.21.E-01 |  | rs4889537 | 9.79.E-02 |
| GPR110 | G protein-coupled receptor 110 | 21 |  | rs13198421 | 2.67.E-02 |  | rs3857591 | 1.94.E-02 |
| HAS1 | hyaluronan synthase 1 | 3 |  | rs12462673 | 9.04.E-02 |  | rs12462673 | 3.27.E-02 |
| HSPD1 | heat shock 60kDa protein 1 (chaperonin) | 2 |  | rs788016 | 7.35.E-01 |  | rs788016 | 6.22.E-01 |
| IL6ST | interleukin 6 signal transducer (gp130, oncostatin M receptor) | 1 |  | rs4865630 | 3.52.E-01 |  | rs4865630 | 4.32.E-01 |
| IQGAP1 | IQ motif containing GTPase activating protein 1 | 20 |  | rs2601188 | 2.28.E-03 |  | rs2601188 | 1.72.E-03 |
| ITGB6 | integrin, beta 6 | 14 |  | rs11901072 | 9.18.E-02 |  | rs11901072 | 1.80.E-01 |
| ITLN1 | intelectin 1 (galactofuranose binding) | 1 |  | rs11265510 | 5.37.E-01 |  | rs11265510 | 4.35.E-01 |
| MAPKAPK2 | mitogen-activated protein kinase-activated protein kinase 2 | 5 |  | rs11119385 | 1.84.E-01 |  | rs11119385 | 2.68.E-01 |
| MAT2A | methionine adenosyltransferase II, alpha | 2 |  | rs2043675 | 1.97.E-01 |  | rs2043675 | 2.56.E-01 |
| MATR3 | matrin 3 | 1 |  | rs10515507 | 1.12.E-02 |  | rs10515507 | 3.35.E-03 |
| MCL1 | myeloid cell leukemia sequence 1 (BCL2-related) | 1 |  | rs3795702 | 6.43.E-01 |  | rs3795702 | 4.60.E-01 |
| MET | met proto-oncogene (hepatocyte growth factor receptor) | 16 |  | rs41752 | 2.35.E-01 |  | rs16945 | 2.12.E-01 |
| **MVD** | **mevalonate (diphospho) decarboxylase** | **1** |  | **rs4420519** | **1.80.E-14** |  | **rs4420519** | **2.92.E-16** |
| MYH11 | myosin, heavy chain 11, smooth muscle | 14 |  | rs3851702 | 1.73.E-02 |  | rs3851702 | 5.16.E-02 |
| NFASC | neurofascin homolog (chicken) | 40 |  | rs4483476 | 1.69.E-02 |  | rs4483476 | 1.92.E-02 |
| NR4A3 | nuclear receptor subfamily 4, group A, member 3 | 5 |  | rs7847479 | 2.85.E-01 |  | rs7847479 | 3.50.E-01 |
| PGS1 | phosphatidylglycerophosphate synthase 1 | 9 |  | rs16971269 | 7.15.E-03 |  | rs16971269 | 2.34.E-03 |
| **PRKG1** | **protein kinase, cGMP-dependent, type I** | **193** |  | **rs7073794** | **1.37.E-04** |  | **rs16925132** | **1.34.E-04** |
| RAB12 | RAB12, member RAS oncogene family | 8 |  | rs12960076 | 6.17.E-02 |  | rs12960076 | 1.22.E-01 |
| RASEF | RAS and EF-hand domain containing | 14 |  | rs1546315 | 8.37.E-03 |  | rs1546315 | 1.80.E-02 |
| RHOB | ras homolog gene family, member B | 3 |  | rs11901246 | 1.64.E-02 |  | rs11901246 | 9.07.E-02 |
| RNF125 | ring finger protein 125 | 1 |  | rs4799626 | 4.81.E-01 |  | rs4799626 | 6.94.E-01 |
| SEC61A1 | Sec61 alpha 1 subunit (S. cerevisiae) | 6 |  | rs3774796 | 9.16.E-02 |  | rs3774796 | 8.52.E-02 |
| SERPINB2 | serpin peptidase inhibitor, clade B, member 2 | 11 |  | rs1015416 | 5.01.E-02 |  | rs9959513 | 5.70.E-02 |
| SERPINE1 | serpin peptidase inhibitor, clade E, member 1 | 4 |  | rs757716 | 3.00.E-01 |  | rs757716 | 2.00.E-02 |
| SFRS2IP | SR-related CTD-associated factor 11 | 3 |  | rs10161306 | 3.89.E-01 |  | rs10161306 | 5.39.E-01 |
| SFRS5 | serine/arginine-rich splicing factor 5 | 4 |  | rs1953399 | 2.92.E-03 |  | rs1953399 | 3.13.E-03 |
| SOD2 | superoxide dismutase 2, mitochondrial | 4 |  | rs8031 | 6.35.E-01 |  | rs6917589 | 3.86.E-01 |
| SRPR | signal recognition particle receptor (docking protein) | 1 |  | rs11220432 | 7.55.E-01 |  | rs11220432 | 6.83.E-01 |
| STC1 | stanniocalcin 1 | 12 |  | rs2060552 | 2.23.E-02 |  | rs2060552 | 7.55.E-03 |
| SUPT16H | suppressor of Ty 16 homolog (S. cerevisiae) | 8 |  | rs10132333 | 3.26.E-01 |  | rs10132333 | 5.15.E-01 |
| TOP1 | topoisomerase (DNA) I | 1 |  | rs12625035 | 9.11.E-01 |  | rs12625035 | 4.40.E-01 |
| TTC3 | tetratricopeptide repeat domain 3 | 16 |  | rs9974286 | 2.34.E-01 |  | rs9974286 | 3.57.E-01 |
| USP10 | ubiquitin specific peptidase 10 | 23 |  | rs16974548 | 6.38.E-02 |  | rs41386649 | 1.52.E-02 |
| WDR1 | WD repeat domain 1 | 26 |  | rs4697926 | 1.14.E-01 |  | rs10516200 | 1.21.E-01 |
| ZNF649 | zinc finger protein 649 | 3 |  | rs1433083 | 1.00.E-01 |  | rs1433083 | 1.11.E-01 |
| ^*^ P-values < 5x10^-4^ was considered genome-wide level significant and marked in bold | | | | | | | | |
