# Supplementary material for: Pathway-Based Analysis Using Genome-wide Association Data from a Korean Non-Small Cell Lung Cancer Study
Source: PLoS One. 2013 Jun 6;8(6):e65396. doi: 10.1371/journal.pone.0065396 (PMC3675130; doi:10.1371/journal.pone.0065396)
Supplement: Table S5 — Comparison of “ABC Transporters” Pathway Genes Between Never-smoker and Ever-smoker. (DOC) [file pone.0065396.s009.doc]

**Table S5. Comparison of "ABC Transporters" Pathway Genes Between Never-smoker and Ever-smoker.**

|  |  |  | **Never Smokers (Additive Model)** | | |  | **Ever Smokers (Additive Model)** | | |
| --- | --- | --- | --- | --- | --- | --- | --- | --- | --- |
| **Gene** | **# of SNPs** |  | **Top SNP** | **P-value** | **OR (95% CI)** |  | **Top SNP** | **P-value** | **OR (95% CI)** |
| ABCA1 | 40 |  | rs2066720 | 3.72 x 10^-2^ | 0.83 (0.69 - 0.99) |  | **rs3905000** | **4.40 x 10^-4^** | **1.81 (1.30 - 2.52)** |
| ABCA2 | 1 |  | rs2049040 | 1.95 x 10^-1^ | 1.15 (0.93 - 1.42) |  | rs2049040 | 8.18 x 10^-1^ | 0.98 (0.79 - 1.21) |
| ABCA3 | 4 |  | rs2014467 | 2.71 x 10^-1^ | 0.90 (0.76 - 1.08) |  | rs150926 | 8.47 x 10^-2^ | 0.86 (0.72 - 1.02) |
| ABCA4 | 37 |  | **rs4147868** | **2.08 x 10^-7^** | **2.65 (1.84 - 3.83)** |  | **rs4147868** | **2.42 x 10^-9^** | **3.39 (2.27 - 5.06)** |
| ABCA5 | 6 |  | rs817126 | 7.24 x 10^-1^ | 1.03 (0.86 - 1.23) |  | rs817126 | 1.22 x 10^-1^ | 1.15 (0.96 - 1.38) |
| ABCA6 | 8 |  | rs8081118 | 1.22 x 10^-1^ | 0.82 (0.64 - 1.05) |  | rs8081118 | 1.82 x 10^-2^ | 0.74 (0.57 - 0.95) |
| ABCA8 | 14 |  | rs4147983 | 3.89 x 10^-2^ | 0.79 (0.62 - 0.99) |  | rs4147983 | 7.82 x 10^-2^ | 0.81 (0.65 - 1.02) |
| ABCA9 | 6 |  | rs11077859 | 4.73 x 10^-1^ | 0.94 (0.78 - 1.12) |  | rs11077858 | 5.39 x 10^-1^ | 0.95 (0.79 - 1.13) |
| ABCA10 | 16 |  | rs7217887 | 3.54 x 10^-1^ | 1.08 (0.92 - 1.28) |  | rs7217887 | 3.30 x 10^-1^ | 1.09 (0.92 - 1.29) |
| ABCA12 | 20 |  | rs17430358 | 2.81 x 10^-2^ | 0.82 (0.69 - 0.98) |  | rs16853009 | 1.73 x 10^-1^ | 0.81 (0.59 - 1.10) |
| ABCA13 | 56 |  | rs2362301 | 1.25 x 10^-2^ | 1.37 (1.07 - 1.74) |  | rs10236551 | 3.35 x 10^-2^ | 1.24 (1.02 - 1.51) |
| ABCB1 | 19 |  | rs4148733 | 6.66 x 10^-2^ | 1.28 (0.98 - 1.66) |  | rs2235047 | 2.51 x 10^-2^ | 1.21 (1.02 - 1.44) |
| ABCB4 | 12 |  | rs2097937 | 3.62 x 10^-2^ | 1.20 (1.01 - 1.43) |  | rs1202283 | 8.15 x 10^-3^ | 0.78 (0.65 - 0.94) |
| ABCB5 | 18 |  | rs11764760 | 1.14 x 10^-1^ | 0.85 (0.69 - 1.04) |  | rs6944093 | 2.10 x 10^-1^ | 0.86 (0.68 - 1.09) |
| ABCB8 | 2 |  | rs2303922 | 6.89 x 10^-1^ | 1.04 (0.86 - 1.25) |  | rs2303922 | 4.39 x 10^-1^ | 1.08 (0.89 - 1.29) |
| ABCB9 | 2 |  | rs4275659 | 4.99 x 10^-1^ | 0.94 (0.79 - 1.13) |  | rs4759359 | 4.58 x 10^-1^ | 1.07 (0.89 - 1.29) |
| ABCB10 | 1 |  | rs10916508 | 2.57 x 10^-1^ | 0.87 (0.69 - 1.11) |  | rs10916508 | 8.07 x 10^-1^ | 0.97 (0.77 - 1.22) |
| ABCB11 | 22 |  | rs2287613 | 2.31 x 10^-1^ | 0.81 (0.57 - 1.14) |  | rs6759156 | 5.51 x 10^-3^ | 1.32 (1.09 - 1.61) |
| ABCC1 | 33 |  | rs12921623 | 2.30 x 10^-2^ | 1.23 (1.03 - 1.47) |  | **rs12921623** | **1.95 x 10^-7^** | **1.67 (1.38 - 2.02)** |
| ABCC2 | 6 |  | rs3740065 | 1.04 x 10^-2^ | 0.80 (0.67 - 0.95) |  | rs2804398 | 4.13 x 10^-2^ | 0.78 (0.61 - 0.99) |
| ABCC3 | 8 |  | rs1978153 | 1.08 x 10^-1^ | 0.85 (0.71 - 1.04) |  | rs739921 | 6.90 x 10^-2^ | 0.75 (0.54 - 1.02) |
| ABCC4 | 64 |  | **rs9524822** | **3.62 x 10^-4^** | **1.93 (1.34 - 2.76)** |  | rs9524822 | 6.71 x 10^-3^ | 1.62 (1.14 - 2.29) |
| ABCC5 | 15 |  | rs1533682 | 2.87 x 10^-2^ | 0.77 (0.61 - 0.97) |  | rs4148594 | 1.39 x 10^-1^ | 1.20 (0.94 - 1.53) |
| ABCC6 | 7 |  | rs4780599 | 6.17 x 10^-2^ | 1.21 (0.99 - 1.48) |  | rs2283508 | 1.71 x 10^-1^ | 1.17 (0.94 - 1.45) |
| ABCC8 | 15 |  | rs2355017 | 6.18 x 10^-2^ | 1.25 (0.99 - 1.57) |  | rs985572 | 6.32 x 10^-3^ | 1.29 (1.08 - 1.56) |
| ABCC9 | 25 |  | rs4148660 | 7.33 x 10^-2^ | 1.19 (0.98 - 1.45) |  | rs10841915 | 3.29 x 10^-2^ | 0.83 (0.70 - 0.99) |
| ABCC10 | 9 |  | rs9357412 | 1.03 x 10^-1^ | 0.80 (0.62 - 1.05) |  | rs9357412 | 1.92 x 10^-1^ | 1.19 (0.92 - 1.54) |
| ABCD2 | 3 |  | rs11172848 | 1.16 x 10^-1^ | 0.82 (0.64 - 1.05) |  | rs11172848 | 1.62 x 10^-1^ | 1.19 (0.93 - 1.53) |
| ABCD3 | 4 |  | rs4148057 | 5.37 x 10^-2^ | 0.77 (0.59 - 1.00) |  | rs1749541 | 3.78 x 10^-2^ | 1.20 (1.01 - 1.43) |
| ABCD4 | 3 |  | rs2074946 | 4.20 x 10^-1^ | 1.17 (0.80 - 1.69) |  | rs2074946 | 5.82 x 10^-1^ | 0.90 (0.63 - 1.30) |
| ABCG1 | 18 |  | rs178744 | 5.09 x 10^-2^ | 1.20 (1.00 - 1.44) |  | rs3787968 | 9.92 x 10^-2^ | 1.17 (0.97 - 1.41) |
| ABCG2 | 11 |  | rs4148157 | 2.17 x 10^-1^ | 1.13 (0.93 - 1.37) |  | rs3109823 | 1.45 x 10^-1^ | 1.17 (0.95 - 1.45) |
| ABCG4 | 1 |  | rs674424 | 9.66 x 10^-1^ | 0.99 (0.74 - 1.33) |  | rs674424 | 5.47 x 10^-1^ | 1.09 (0.82 - 1.45) |
| ABCG5 | 4 |  | rs4131229 | 5.98 x 10^-1^ | 0.95 (0.77 - 1.17) |  | rs2278357 | 9.01 x 10^-3^ | 1.42 (1.09 - 1.85) |
| ABCG8 | 3 |  | rs4148202 | 2.44 x 10^-1^ | 0.88 (0.71 - 1.09) |  | rs4148206 | 4.74 x 10^-1^ | 1.09 (0.87 - 1.36) |
| CFTR | 13 |  | rs213987 | 6.12 x 10^-2^ | 0.84 (0.70 - 1.01) |  | rs2283054 | 5.54 x 10^-2^ | 0.85 (0.71 - 1.00) |
| TAP1 | 2 |  | rs4711312 | 3.58 x 10^-1^ | 0.89 (0.70 - 1.14) |  | rs12529313 | 3.56 x 10^-1^ | 1.10 (0.90 - 1.36) |
| TAP2 | 6 |  | rs1800454 | 2.39 x 10^-1^ | 0.86 (0.67 - 1.10) |  | rs241429 | 1.30 x 10^-1^ | 0.87 (0.72 - 1.04) |
| ^*^ P-values<5x10^-4^ was considered genome-wide level significant and marked in bold | | | | | | | | | |
